# Fatty Acid Amide Hydrolase (FAAH) Inhibitors Exert Pharmacological Effects, but Lack Antinociceptive Efficacy in Rats with Neuropathic Spinal Cord Injury Pain

**DOI:** 10.1371/journal.pone.0096396

**Published:** 2014-05-02

**Authors:** Aldric T. Hama, Peter Germano, Matthew S. Varghese, Benjamin F. Cravatt, G. Todd Milne, James P. Pearson, Jacqueline Sagen

**Affiliations:** 1 Miami Project to Cure Paralysis, University of Miami Miller School of Medicine, Miami, Florida, United States of America; 2 Ironwood Pharmaceuticals, Inc., Cambridge, Massachusetts, United States of America; 3 Department of Chemical Physiology, The Skaggs Institute for Chemical Biology, The Scripps Research Institute, La Jolla, California, United States of America; Nathan Kline Institute for Psychiatric Research and New York School of Medicine, United States of America

## Abstract

Amelioration of neuropathic spinal cord injury (SCI) pain is a clinical challenge. Increasing the endocannabinoid anandamide and other fatty acid amides (FAA) by blocking fatty acid amide hydrolase (FAAH) has been shown to be antinociceptive in a number of animal models of chronic pain. However, an antinociceptive effect of blocking FAAH has yet to be demonstrated in a rat model of neuropathic SCI pain. Four weeks following a SCI, rats developed significantly decreased hind paw withdrawal thresholds, indicative of below-level cutaneous hypersensitivity. A group of SCI rats were systemically treated (i.p.) with either the selective FAAH inhibitor URB597 or vehicle twice daily for seven days. A separate group of SCI rats received a single dose (p.o.) of either the selective FAAH inhibitor PF-3845 or vehicle. Following behavioral testing, levels of the FAA *N-*arachidonoylethanolamide, *N-*oleoyl ethanolamide and *N-*palmitoyl ethanolamide were quantified in brain and spinal cord from SCI rats. Four weeks following SCI, FAA levels were markedly reduced in spinal cord tissue. Although systemic treatment with URB597 significantly increased CNS FAA levels, no antinociceptive effect was observed. A significant elevation of CNS FAA levels was also observed following oral PF-3845 treatment, but only a modest antinociceptive effect was observed. Increasing CNS FAA levels alone does not lead to robust amelioration of below-level neuropathic SCI pain. Perhaps utilizing FAAH inhibition in conjunction with other analgesic mechanisms could be an effective analgesic therapy.

## Introduction

In addition to motor and visceral dysfunction, a serious consequence of spinal cord injury (SCI) is chronic pain. It is estimated that 65% of SCI patients suffer from chronic pain, with about one-third of these patients rating their pain as severe [1]. Although pain can occur above and at the level of the spinal lesion, symptoms with similarity to peripheral neuropathic pain can occur below the spinal lesion [2]. Also, below-level neuropathic pain is particularly challenging to treat in that adverse side effects of commonly prescribed neuropathic pain analgesics may exacerbate existing dysfunctions, such as urinary retention with antidepressants and constipation with opioids. Thus, it is crucial that novel therapeutics be developed for this particular patient population.

A number of studies have shown that activation of the cannabinoid (CB) receptor leads to a robust antinociception in a variety of preclinical pain models [3]. The antinociceptive effects of nonselective CB receptor agonists in rats are blocked with either cannabinoid receptor type-1 (CB_1_) or cannabinoid receptor type-2 (CB_2_) receptor antagonists. Limited clinical evidence and SCI patient survey suggests that CB receptor activation with CB ligands derived from *Cannabis sativa* reduces SCI pain [4], [5]. In humans, the psychological and physiological effects of CB agonists are blocked by treatment with the CB_1_ receptor antagonist rimonabant (SR141719A) indicating the importance of CB_1_ receptors in mediating the effects of CB [6].

Intrathecal administrations of fatty acid amide (FAA) endocannabinoids, such as *N-*arachidonoylethanolamide (AEA), demonstrate robust antinociception in models of tissue injury-induced cutaneous hypersensitivity [7], [8]. One method of enhancing CNS levels of endocannabinoids and other antinociceptive non-cannabinoid FAA, such as *N-*oleoyl ethanolamide (OEA) and *N-*palmitoyl ethanolamide (PEA), is by blocking one of their catabolic enzymes, fatty acid amide hydrolase (FAAH) [9], [10]. Systemic as well as intrathecal administration of selective FAAH inhibitors have demonstrated significant antinociception in preclinical models of peripheral neuropathic pain [3], [9], [11]. The antinociceptive effects positively correlated with increased CNS endocannabinoid levels and were blocked with administration of either CB_1_ or CB_2_ receptor antagonist [12], [13]. Even though the antinociceptive effect is mediated by CB_1_ receptors, there is a lack of side effects with FAAH inhibition commonly associated with CB_1_ receptor agonists, including hypothermia, catalepsy and decreased locomotor activity [14]. It is possible that a part of the antinociceptive effect of increased CNS FAA is due to a non-CB receptor mediated mechanism, such activity at the transient receptor potential vanilloid receptor (TRPV1) channel [11].

Acute SCI in rats can lead to persistent neuropathic pain-like symptoms [15]–[17]. Robust cutaneous hypersensitivity of the hind paws to both noxious and innocuous stimuli is obtained following spinal compression [18]. A number of clinical analgesics do not display efficacy in SCI rats despite demonstrating efficacy in other neuropathic pain models [17], [18]. However, treatment with a non-subtype selective CB receptor agonist WIN 55,212-2 led to a significant antinociception which was reversed by treatment with rimonabant indicating that therapeutic efficacy was mediated by CB_1_ receptors [19]. Furthermore, no tolerance to the antinociceptive effect of WIN 55,212-2 was observed over a seven-day treatment period, whereas morphine efficacy gradually diminished during the same treatment period [20]. While exogenous CB_1_ receptor agonists may hold promise as analgesics for neuropathic SCI pain, in addition to target-mediated side effects, their current legal status and difficulty in “blinding” the psychotropic effects of cannabinoids make clinical development controversial [21]. Although antinociceptive efficacy of FAAH inhibitors has been observed in various models of peripheral tissue injury-induced pain, efficacy has yet to be demonstrated in neuropathic SCI pain.

The goal of the current study is to evaluate the effect of blocking FAAH with the inhibitors URB597 and PF-3845 on below-level cutaneous hypersensitivity SCI rats. URB597 is a phenyl carbamate inhibitor and PF-3845 is a piperidine urea FAAH inhibitor [9] which irreversibly bind to FAAH. Most previous studies of URB597 demonstrated antinociception following a single dose, but one study showed that repeated dosing over time was needed to attain significant efficacy [22]. Thus, in the current study, SCI rats were systemically treated with URB597 over a seven-day period. Following the treatment and testing period, brain and spinal cord were collected for measurement of FAA by liquid chromatography-tandem mass spectrometry (LC-MS/MS) analysis. As a comparator, WIN 55,212-2 was tested in parallel with FAAH inhibitors in SCI rats. Whereas treatment with WIN 55,212-2 resulted in marked antinociception, there was a lack of comparable antinociception following FAAH treatment in SCI rats.

## Materials and Methods

### Animals

All procedures were reviewed and approved by the University of Miami Animal Care and Use Committee (Protocol No. 07-134) and followed the recommendations within the *Guide for the Care and Use of Laboratory Animals* (National Research Council). Every effort was made to use the least possible number of animals and to minimize pain and distress. Male Sprague-Dawley rats (130–150 g at the time of surgery; Harlan, Indianapolis, IN) were housed two per cage and allowed free access to food and water before and after surgery. Lighting was on a 12 hr light/dark cycle. Temperature and humidity were controlled to 22±1°C and 50±10%, respectively. Rats were acclimated to the animal facility for at least 5 days prior to use.

### General experimental plan

Baseline hind paw withdrawal thresholds and locomotor function were assessed before and 4 weeks following SCI surgery. Spinal cord injured rats were dosed beginning 4 weeks after surgery for seven days. Although SCI rats were dosed daily, withdrawal thresholds were measured only on the first, third and seventh day of dosing (i.e. 28, 30 and 35 days post-SCI surgery). To assess a possible effect of daily compound treatment on locomotor function, locomotor rating scores were obtained from SCI rats 4 weeks after surgery, prior to compound administration, and on the sixth day of compound treatment (i.e. 34 days post-SCI surgery).

### Spinal cord injury in the rat

Aseptic surgical technique and sterile instruments were used. The procedure to induce an acute SCI has been previously described [18]. Rats were anesthetized and maintained with isoflurane in O_2_ for the duration of the surgery. The back was shaved and the skin cleaned with chlorhexadine. A laminectomy was performed to expose spinal segment T6-T7. A microvascular clip (20 g compressive force, Harvard Apparatus, Hollister, MA) was placed vertically on the exposed thoracic spinal cord, such that clip compressed the entire segment, and then left in place for 60 seconds. Care was taken not to cut the dura or disturb nearby spinal nerve roots. Following spinal compression, the clip was removed, the muscles sutured shut and the skin closed with wound clips. Urinary bladders were expressed twice daily until voiding was regained. Spontaneous voiding returned about 1 or 2 days following spinal compression.

### Hind paw withdrawal threshold

Prior to spinal compression surgery, hind paw withdrawal thresholds were measured. The up-down method was used to quantify hind paw responsiveness to innocuous tactile stimulation [23]. Rats were placed in clear Plexiglas containers resting on an elevated wire mesh floor and allowed to acclimate to the chamber for at least 30 min. Eight specific calibrated von Frey filaments were used to determine the withdrawal threshold (0.4, 0.6, 1, 2, 4, 6, 8, 15 grams; Stoelting Co., Woodale, IL). The filament was applied perpendicularly the plantar skin of the hind paw, bowed slightly and held in place for six seconds. A hind paw withdrawal from the filament led to testing with a lower force filament. A lack of response led to the use of the next higher force filament. From the pattern of responses to the filaments, a withdrawal threshold was calculated. Both hind paws were tested and about 5 min separated testing of the opposite paw. An upper withdrawal threshold of 15 g was used. Prior to surgery, rat withdrawal thresholds were 15 g. In order for SCI rats to be included in the study, withdrawal thresholds of one paw needed to be 4 g or less.

### Locomotor function: Basso, Beattie, and Bresnahan Score

Following threshold measurement, below-level motor functionality was assessed using a standardized “Locomotor Rating Scale” devised by Basso, Beattie, and Bresnahan (BBB) [24]. The scale ranges from 0, no hind limb function, to 21, normal, coordinated movement of the hind limbs as observed in uninjured rats. Rats were placed in a 1.2 m diameter arena and allowed to continuously move within a four minute observation period. Two observers rated hind limb function, such as movement at joints, hind paw stepping and fore paw-hind paw coordination. Following the observation period, the scores from both hind limbs were averaged to give a final BBB score. Prior to surgery, BBB scores of naive rats are 21. Spinal cord injured-rats were rated one day prior to the beginning of the treatment period and reassessed on the sixth day of treatment following determination of withdrawal thresholds.

### Pharmacological treatments

Following baseline behavioral assessment in SCI rats, rats were divided into four treatment groups. Spinal cord-injured rats were injected with either URB597 (i.p.), WIN 55,212-2 (s.c.) or vehicle (i.p.) twice daily (at approximately 900 and 1700) for 7 days. Withdrawal thresholds were measured following the first daily injection. Following injection, rats were tested once every 30 minutes for up to four hours post-injection. On the sixth day of treatment, following final determination of withdrawal threshold, BBB scores were determined. On the seventh day, immediately following testing at four hours post-injection, rats were deeply anesthetized with isoflurane, decapitated and brain and spinal cord tissues were collected.

In a separate group of SCI rats, either PF-3845 or vehicle was orally administered and rats were tested once every hour up to four hours post-administration. Because of stress associated with p.o. dosing in SCI rats, rats were dosed only once and not treated over a seven-day period. At the last testing time point, rats were deeply anesthetized with isoflurane, decapitated and brain and spinal tissues were collected. Uninjured age-matched rats were also treated with either PF-3845 or vehicle (p.o.) and tissues were collected at the same time as the SCI rats. At the same time, age-matched uninjured rats were treated with either URB597 (i.p.), WIN 55,212-2 (s.c.), or vehicle (i.p.), were deeply anesthetized, decapitated and tissues were collected four hours post-treatment to determine baseline FAA levels and to determine the acute effects of the compounds on FAA levels in uninjured rats. An additional group of uninjured rats were treated with URB597 (i.p.) or vehicle (i.p.), euthanized and brain and spinal cord tissues were collected two hours after treatment.

The spinal cord was divided into four segments: lumbar (L4/L5), a 1 cm segment rostral to the compression epicenter, a 1 cm segment of the epicenter and a 1 cm segment caudal to the epicenter. Tissues were flash frozen on dry ice and stored at −80°C until assay.

### Assessment of brain and spinal cord levels of fatty acid amides by liquid chromatography-tandem mass spectrometry

Brain samples were prepared using a method adapted from previously published methods [25]. Briefly, brain samples were removed from −80°C freezer and placed on dry ice. Individual brains were transferred to a clean, tared 50 ml capacity polypropylene conical tube and weights were recorded. A solution of ethyl acetate∶hexanes (9∶1) was immediately added to each conical tube along with a FAA standard molecule (Palmitoyl Propanolamide, Ironwood Pharmaceuticals, Inc., Cambridge, MA). Samples were homogenized for 15 seconds using an electric-powered mechanical tissue disrupter (Omni Prep Multi-Sample Homogenizer Part Number: 06-021, Omni International, Kennesaw, GA) fitted with stainless steel probe (10 mm×110 mm Stainless Steel Omni Prep/THQ Homogenizer Probe, Omni International, Kennesaw, GA) washed with approximately 30% water, and homogenized for another 15 seconds. Samples were vortexed and centrifuged (2,000×g for 30 minutes at 10°C). After centrifugation, the upper organic layer was recovered and the samples were evaporated to dryness under nitrogen gas. Samples were not subjected to solid phase extraction. After reconstitution in 1 ml chloroform∶methanol (1∶3), samples were centrifuged (16,000×g for 3 minutes at room temperature) to sediment any particulates. A 100 µl aliquot of each sample supernatant was transferred to individual wells on a 96 well plate. Each sample was diluted 1∶1 with methanol containing the internal standard d4-AEA (arachidonoyl-ethanolamide) labeled with 4 deuterium atoms replacing the 4 H's on the methylenes of ethanolamine moiety, Cayman Chemical, Ann Arbor, MI) Waters Acquity/TQD system in positive ion (ES+) mode. Samples were maintained at 6°C.

The procedure for FAA extraction from spinal cord tissue segments was similar to brain FAA extraction procedure listed above with the following change. After evaporation of organic solvents from tissue homogenates the residue from extraction was dissolved in 0.25 ml chloroform∶methanol (1∶3) instead of 1 ml volume used for brain extracts.

### Liquid chromatography-tandem mass spectrometry conditions

Liquid chromatography was conducted using a Higgins Clipeus HPLC column [C8 reverse phase, dimensions 2.1×30 mm, particle size 5 micron (Mountain View, CA)] and samples were eluted using a gradient employing 2 mobile phases A and B from 30%–95% B [HPLC mobile phases A (5 mM ammonium acetate in water) and HPLC mobile phase B (5 mM ammonium acetate in 85∶10∶5 acetonitrile: isopropanol: water)] using a flow rate of 0.4 ml/min. MS/MS. The following mass transitions were monitored AEA: 348.2>62 m/z, OEA: 326.2>62 m/z, PEA: 300.2>62 m/z, Palmitoyl Propanolamide: 314.2>75.9 m/z, D4-AEA: 352.2>66 m/z (last two are internal standards for fatty acid amides). Chromatograms were integrated and quantified by response to the internal standard peak area using Quanlynx software V4.0 SP4 (Micromass, Ltd).

### Compound treatments

URB597 (3 mg/kg, Cayman Chemicals, Ann Arbor, MI) was dissolved in 10% dimethyl sulfoxide (DMSO): 10% Cremophor EL: 80% saline and administered i.p. in a volume of 1.5 ml/kg. URB597 was prepared daily, prior to dosing. WIN 55,212-2 mesylate (3 mg/kg, Sigma-Aldrich Co., St. Louis, MO) was dissolved in 45% (2-Hydroxypropyl)-β-cyclodextrin (Sigma-Aldrich, Co.) in saline and administered s.c. in a volume of 2 ml/kg. PF-3845 (3, 10 mg/kg, synthesized as described by Ahn et al., 2009 b was dissolved in 10% DMSO: 10% Cremophor EL: 80% saline and administered p.o. in a volume of 5 ml/kg.

### Statistical analysis

**Figure 1:**
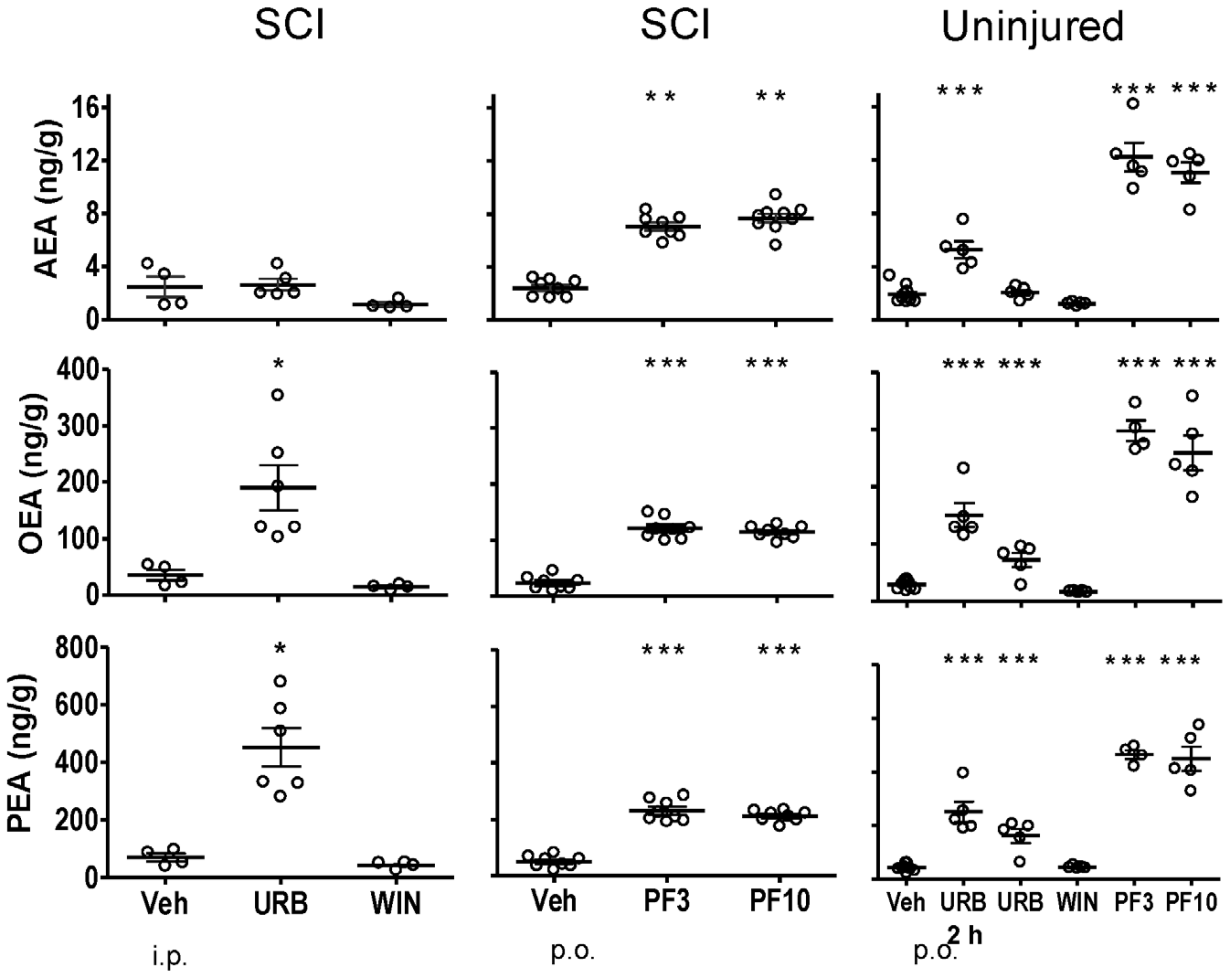
Fatty acid amide concentrations in brain following treatment with FAAH inhibitors, WIN 55,212-2 and vehicle in SCI and uninjured rats. Rats were treated with either URB597 (URB; 3 mg/kg, i.p.), WIN 55,212-2 (WIN; 3 mg/kg, s.c.) or vehicle (Veh; 1.5 ml/kg, i.p.) for seven days and euthanized four hours following the last treatment. One group of URB597 rats were euthanized two hours (2 h) following a single treatment. Rats that received PF-3845 (PF3, PF10; 3, 10 mg/kg, p.o) or vehicle (Veh, p.o.) were treated once and euthanized four hours following dosing. Levels of AEA, OEA and PEA from each rat are shown, the thick horizontal line is the mean and the thin horizontal lines are the S.E.M. n = 4–10/group. * *p*<0.05, **p<0.01, ***p<0.001 vs. vehicle (Student's t-test).

**Figure 2:**
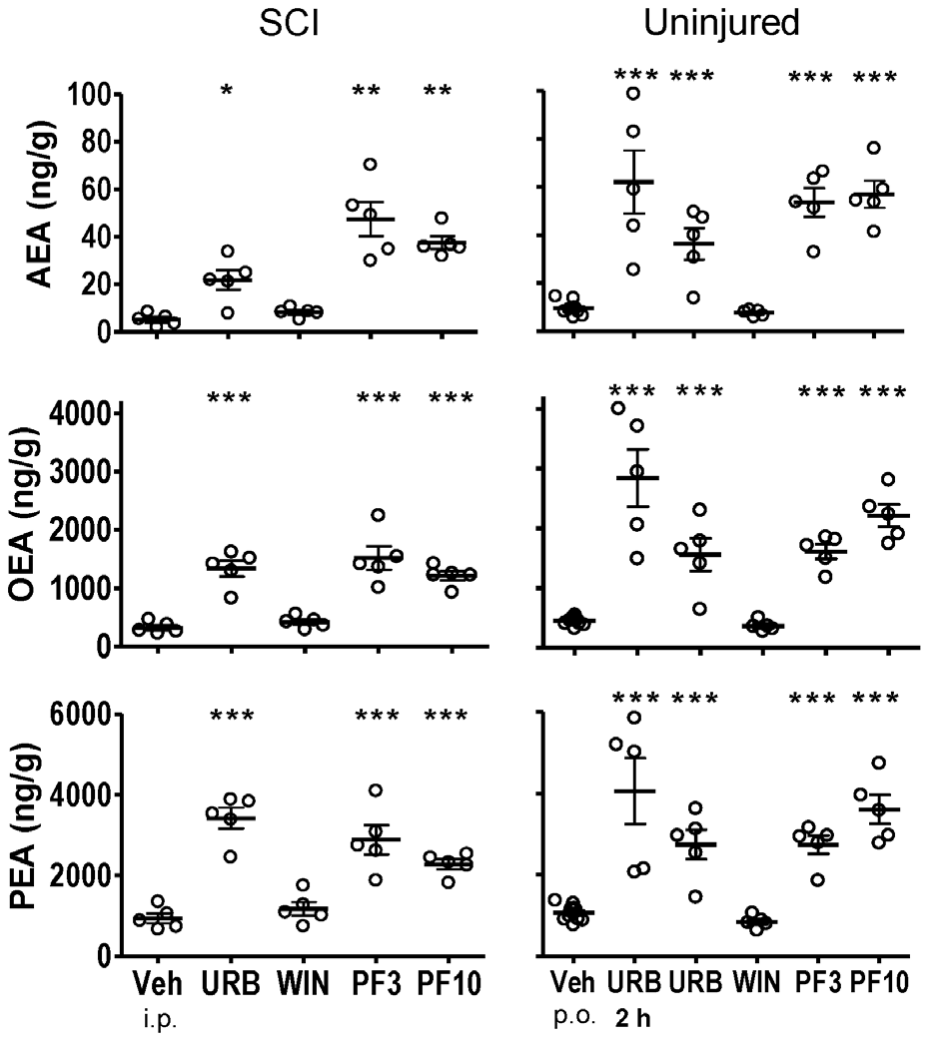
Fatty acid amide concentrations in thoracic spinal cord segments rostral to the spinal injury from SCI rats or comparable thoracic spinal cord segments from uninjured rats following treatment with FAAH inhibitors, WIN 55,212-2 and vehicle. Both SCI and uninjured rats were treated with either URB597 (URB; 3 mg/kg, i.p.), WIN 55,212-2 (WIN; 3 mg/kg, s.c.) or vehicle (Veh; 1.5 ml/kg, i.p.) for seven days and euthanized four hours following the last treatment. Rats that received PF-3845 (PF3, PF10) were treated once (3, 10 mg/kg, p.o.) and euthanized four hours following dosing. One group of uninjured rats was treated once with URB597 and euthanized two hours (2 h) following treatment. The FAA levels of thoracic spinal cord of uninjured rats from this figure are repeated for Figures 3 and 4. Levels of AEA, OEA and PEA from each rat are shown, the thick horizontal line is the mean and the thin horizontal lines are the S.E.M. n = 4–10/group. * *p*<0.05, **p<0.01, ***p<0.001 vs. vehicle (Student's t-test).

**Figure 3:**
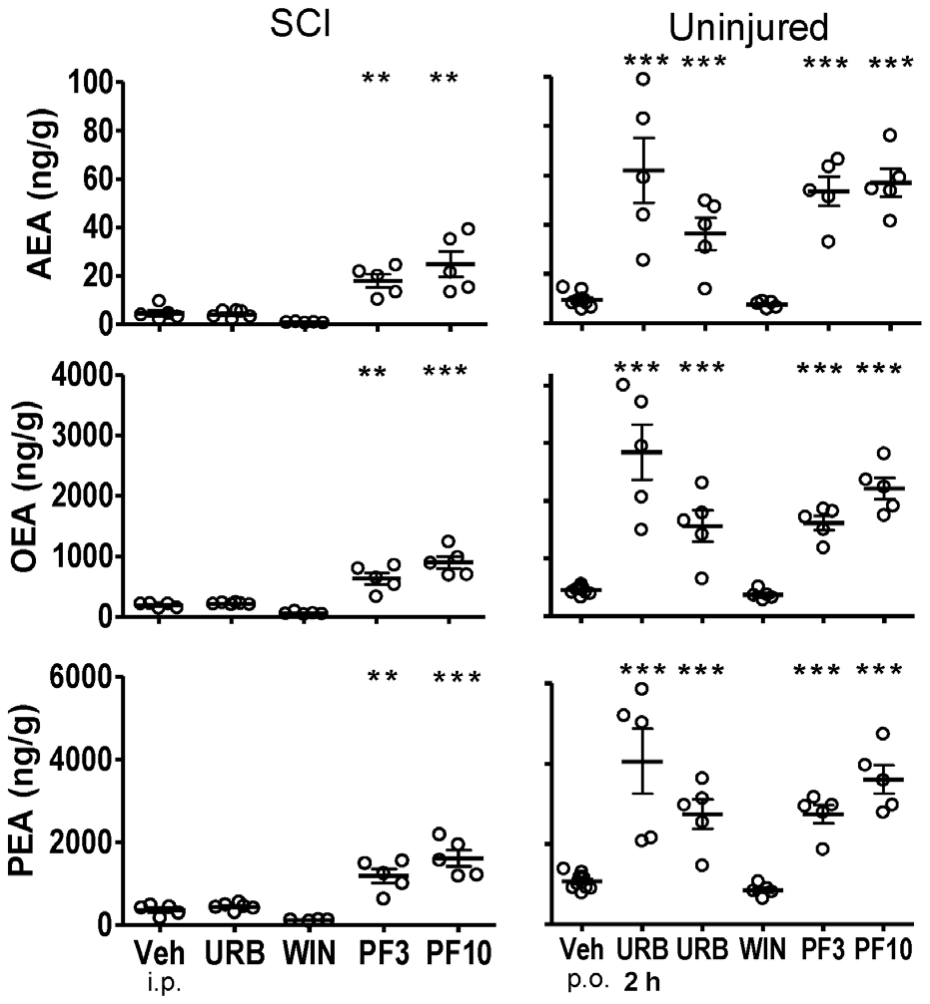
Fatty acid amide concentrations in thoracic spinal cord segments from the epicenter of injury from SCI rats or comparable spinal cord segments from uninjured rats following treatment with FAAH inhibitors, WIN 55,212-2 and vehicle. Both SCI and uninjured rats were treated with either URB597 (URB; 3 mg/kg, i.p.), WIN 55,212-2 (WIN; 3 mg/kg, s.c.) or vehicle (Veh; 1 ml/kg, i.p.) for seven days and euthanized four hours following the last treatment. One group of uninjured rats was treated once with URB597 and euthanized two hours (2 hr) following treatment. Rats that received PF-3845 (PF3, PF10) were treated once (3, 10 mg/kg, p.o.) and euthanized four hours following dosing. Levels of AEA, OEA and PEA from each rat are shown, the thick horizontal line is the mean and the thin horizontal lines are the S.E.M. n = 4–10/group. * *p*<0.05, **p<0.01, ***p<0.001 vs. vehicle (Student's t-test).

**Figure 4:**
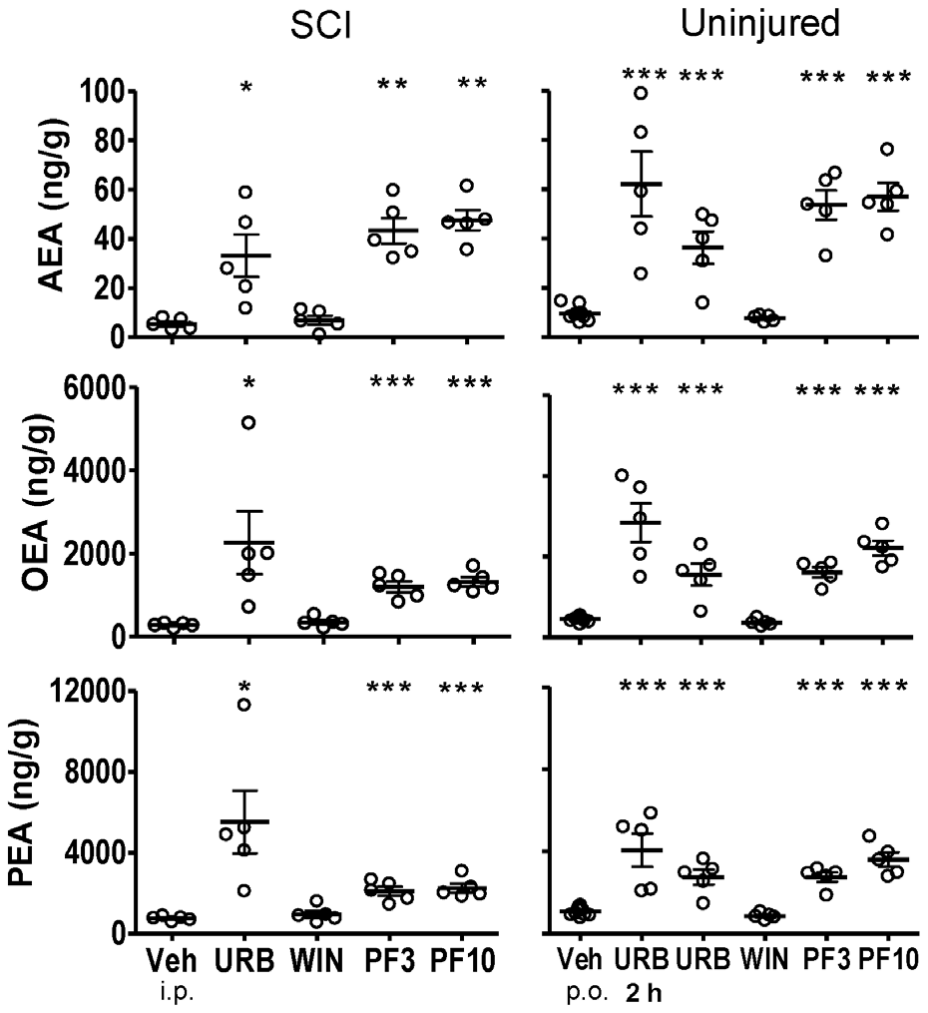
Fatty acid amide concentrations in thoracic spinal cord segments caudal to the spinal injury from SCI rats or comparable spinal cord segments from uninjured rats following treatment with FAAH inhibitors, WIN 55,212-2 and vehicle. Rats were treated with either URB597 (URB; 3 mg/kg, i.p.), WIN 55,212-2 (WIN; 3 mg/kg, s.c.) or vehicle (Veh; 1 ml/kg, i.p.) for seven days and euthanized four hours following the last treatment. One group of uninjured rats was treated once with URB597 and euthanized two hours (2 h) following treatment. Rats that received PF-3845 (PF3, PF10) were treated once (3, 10 mg/kg, p.o.) and euthanized four hours following dosing. Levels of AEA, OEA and PEA from each rat are shown, the dark horizontal line is the mean and the light horizontal lines are the S.E.M. n = 4–10/group. * *p*<0.05, **p<0.01, ***p<0.001 vs. vehicle (Student's t-test).

**Figure 5:**
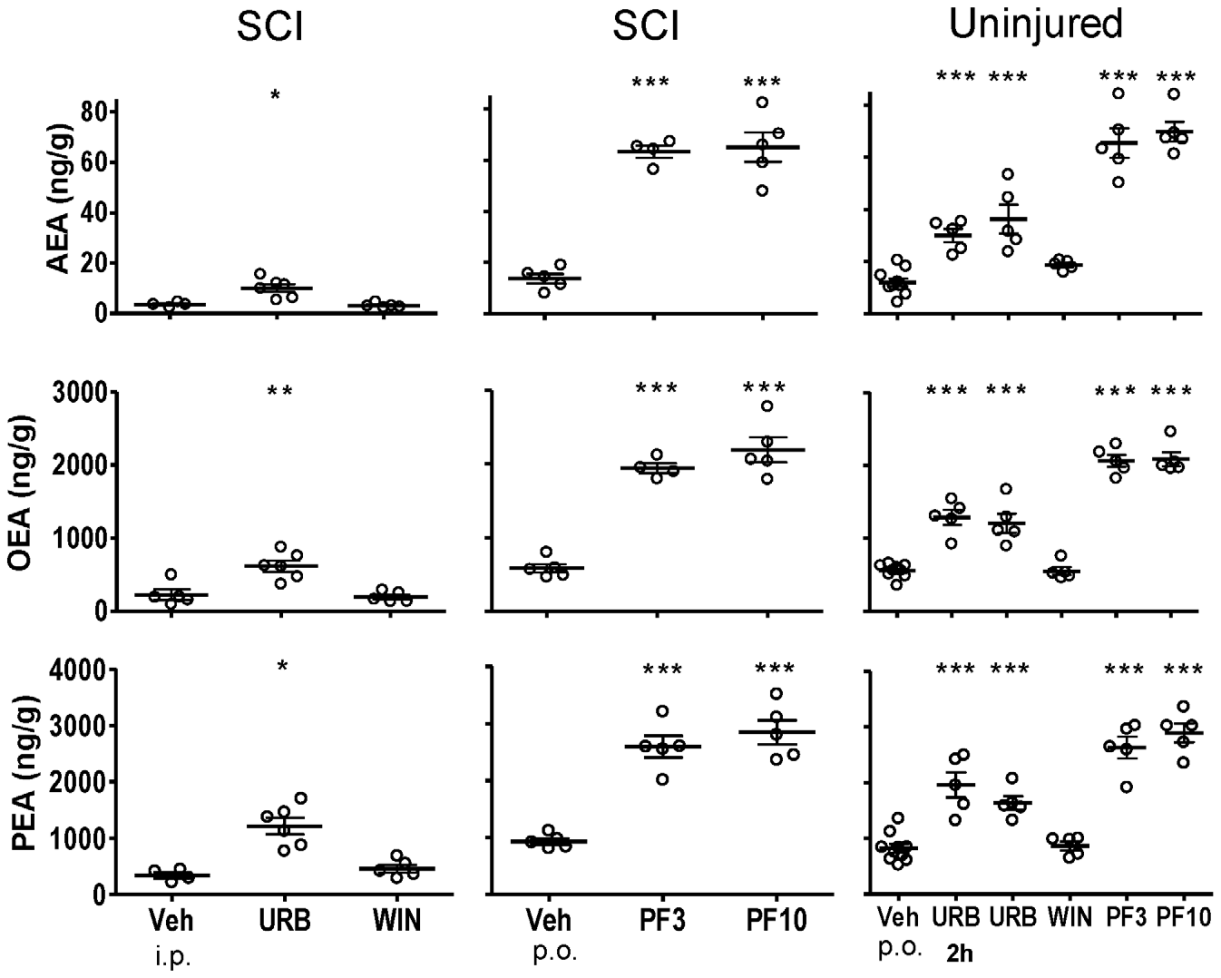
Fatty acid amide concentrations in lumbar spinal cord from SCI rats or uninjured rats following treatment with FAAH inhibitors, WIN 55,212-2 and vehicle. Rats were treated with either URB597 (URB; 3 mg/kg, i.p.), WIN 55,212-2 (WIN; 3 mg/kg, s.c.) or vehicle (Veh; 1.5 ml/kg, i.p.) for seven days and euthanized four hours following the last treatment. One group of uninjured rats was treated once with URB597 and euthanized two hours (2 h) following treatment. Rats that received PF-3845 (PF3, PF10) were treated once (3, 10 mg/kg, p.o.) and euthanized four hours following dosing. Levels of AEA, OEA and PEA from each rat are shown, the thick horizontal line is the mean and the thin horizontal lines are the S.E.M. n = 4–10/group. * *p*<0.05, **p<0.01, ***p<0.001 vs. vehicle (Student's t-test).

Analyses of LC-MS/MS data were conducted using GraphPad Prism software. For FAA, statistical comparisons between treatment groups were made using an unpaired, two-tailed Student's *t*-test. To determine fold-change in levels of FAA, the levels were normalized to the respective vehicle-treated group. *p* values less than 0.05 were considered significant. To assess the effect of treatment over time on behaviors, a two-way repeated measures analysis of variance (ANOVA) was performed with Student-Newman-Keuls for post hoc analysis. Fatty acid amide levels from each rat were plotted (Fig. 1–5) and the mean ± S.E.M. were calculated. Behavioral data are presented as mean ± S.E.M.

## Results

### Effect of spinal cord injury on CNS FAA levels

Table 1 summarizes changes in CNS FAA levels four weeks after SCI. The most apparent changes in SCI tissue, compared to uninjured tissues, are of decreased levels of OEA and PEA of thoracic spinal cord at and near the epicenter.

**Table 1:** Summary of Changes in Fatty Acid Amides Four Weeks Following a Spinal Cord Injury in Rats.

	AEA	OEA	PEA
Brain	n.c.	n.c.	n.c.
Spinal cord			
Rostral to epicenter	↓ n.s.	↓*	↓*
Epicenter	↓ n.s.	↓*	↓*
Caudal to epicenter	↓ n.s.	↓*	↓*
Lumbar spinal cord	↓ n.s.	↓ n.s.	↓ n.s.

Changes in fatty acid amides (AEA, *N*-arachidonoylethanolamide; OEA, *N*-oleoyl ethanolamide; PEA, *N*-palmitoyl ethanolamide) four weeks following spinal cord injury compared to that of uninjured rats.

↓, decrease; n.c., no change; n.s., not statistically significant; *p<0.05 (Student's t-test).

#### Whole Brain

In untreated, uninjured rats, the mean brain levels of AEA, OEA and PEA were 2.2±0.51, 33.7±3.8 and 67.7±5.0 ng/g tissue, respectively (Fig. 1). In SCI vehicle-treated rats, the mean brain levels of AEA, OEA and PEA were 2.5±0.8, 35.6±9.3 and 69.4±13.6 ng/g tissue, respectively. Brain FAA levels from vehicle-treated SCI rats were not significantly different from brain levels from uninjured rats (*p*>0.05).

#### Thoracic Spinal Cord

Spinal cord injury led to marked decreases in OEA and PEA content in spinal tissues. The mean level of OEA in thoracic spinal cord from uninjured rats was 652.1±99.1 ng/g tissue. The mean levels of OEA in tissue rostral to the epicenter, the epicenter and caudal to the epicenter from SCI rats were 318.6±44.1, 188.8±17.9 and 275.3±22.3 ng/g tissue, respectively (*p*<0.05, rostral, epicenter and caudal vs. uninjured OEA; Fig. 2–4). The mean level of PEA in thoracic spinal cord from uninjured rats was 1108.0±148.7 ng/g tissue. The mean level of PEA in tissue rostral to the epicenter, the epicenter and caudal to the epicenter from SCI rats were 941.4±122.3, 351.5±56.5 and 740.2±53.2 ng/g tissue (e.g. Fig. 3; *p*<0.05, epicenter and caudal vs. uninjured PEA).

By contrast, mean levels of AEA in spinal tissues from SCI rats were not significantly changed from that of uninjured rats. Levels of AEA in the rostral, epicenter and caudal spinal segments from SCI rats were 5.1±1.1, 4.6±1.3 and 5.4±1.0 ng/g tissue, respectively. Levels of AEA in comparable spinal segments from uninjured rats were 6.0±1.3, 8.0±1.9 and 6.2±0.4 ng/g tissue, respectively. While there was a trend of decreased levels of AEA in spinal tissues from SCI rats, this was not statistically significant from that of uninjured rats (*p*>0.05).

#### Lumbar Spinal Cord

In lumbar spinal cord from untreated, uninjured rats, the mean levels of AEA, OEA and PEA were 3.8±0.4, 241.0±32.8 and 493.4±61.5 ng/g tissue, respectively. In lumbar spinal cord from SCI rats, the mean levels of AEA, OEA and PEA were 2.6±0.1, 224.1±30.0 and 344.7±53.8 ng/g tissue, respectively. The levels of FAA in lumbar spinal cord from SCI rats, while slightly decreased, were not significantly different from that of uninjured rats (*p*>0.05; Fig. 5).

### Effect of FAAH inhibitors and WIN 55,212-2 on CNS FAA levels

#### URB597

Four hours post-treatment with URB597, levels of AEA, OEA and PEA were significantly increased in spinal segments rostral (Fig. 2) and caudal (Fig. 4) to the injury and lumbar segment (Fig. 5) of spinal cord from SCI rats (*p*<0.05 vs. vehicle treatment). Levels of AEA were unchanged in brains from both uninjured and SCI rats, despite clear increases in OEA and PEA (Fig. 1). To confirm that the dose of URB597 was effective in blocking brain FAAH, uninjured rats were euthanized two hours following URB597 treatment; FAA were significantly increased (*p*<0.05 vs. vehicle; Fig. 1). In contrast to the increases in FAA rostral and caudal to the spinal injury, there was a lack of effect of URB597 treatment at the epicenter four hours after treatment (*p*>0.05 vs. vehicle treatment, Fig. 3).

Table 2 summaries changes in CNS FAA content following URB597 treatment in SCI and uninjured rats. The effects of URB597 treatment, as fold-change over vehicle-treatment, on CNS FAA are summarized following a seven day treatment schedule and following a single treatment.

**Table 2:** Fold-change of tissue fatty acid amide levels following treatment with URB597 or WIN 55,212-2 relative to vehicle treatment.

**Seven days**		AEA					OEA					PEA				
Rats	Treatment	Brain	Rostral	Epicenter	Caudal	Lumbar	Brain	Rostral	Epicenter	Caudal	Lumbar	Brain	Rostral	Epicenter	Caudal	Lumbar
SCI	Vehicle	1	1	1	1	1	1	1	1	1	1	1	1	1	1	1
SCI	URB	1.6 (*)	4.3 (**)	0.9 (n.s.)	6.1 (*)	2.9 (*)	5.3 (*)	4.2 (***)	1.2 (n.s.)	8.2 (*)	2.8 (**)	6.5 (***)	3.6 (***)	1.2 (n.s.)	7.5 (*)	3.5 (*)
SCI	WIN	0.5 (n.s.)	1.6 (n.s.)	0.2 (n.s)	1.3 (n.s.)	0.9 (n.s.)	0.4 (n.s.)	1.3 (n.s.)	0.3 (n.s.)	1.2 (n.s.)	0.9 (n.s.)	0.6 (n.s.)	1.3 (n.s.)	0.3 (n.s.)	1.3 (n.s.)	1.3 (n.s.)
**One day**																
N.I.	Vehicle	1	—	1	—	1	1	—	1	—	1	1	—	1	—	1
N.I.	URB (2 h)	2.7 (*)	—	6.5 (***)	—	2.5 (***)	5.1 (***)	—	6.3 (***)	—	2.3 (***)	5.7 (***)	—	3.8 (***)	—	2.3 (***)
N.I.	URB	1.1 (n.s.)	—	3.8 (***)	—	3.0 (***)	2.5 (***)	—	3.5 (***)	—	2.2 (***)	3.6 (***)	—	2.6 (***)	—	2.0 (***)
N.I.	WIN	0.6 (n.s.)	—	0.8 (n.s.)	—	1.6 (n.s)	0.6 (n.s.)	—	0.8 (n.s.)	—	1 (n.s.)	1 (n.s.)	—	0.8 (n.s.)	—	1 (n.s.)

Values are fold-changes compared to vehicle treatment levels in spinal cord injured (SCI) and non-injured (N.I.) rats. On the seventh day of treatment, tissues were collected 4 hours post-administration with either vehicle, URB597 (URB, 3 mg/kg, i.p.) or WIN 55,212-2 (WIN, 3 mg/kg, s.c.) from SCI rats. Non-injured rats were treated once and tissues collected four hours post-treatment. In one set of non-injured rats (n = 5), tissues were collected two hours (2 h) post-treatment with URB597.

“Epicenter” refers to thoracic spinal cord tissue either from the site of injury from SCI rats or comparable spinal cord tissue from non-injured rats.

n.s., not significant.

*p<0.05, **p<0.01, ***p<0.001 vs. vehicle-treated (Student's t-test).

#### PF-3845

PF-3845 also consistently increased CNS FAA levels in both uninjured and SCI rats. However, unlike URB597, PF-3845 also elevated FAAs at the epicenter in this experiment, at both doses, four hours after treatment (*p*<0.05 vs. vehicle; Fig. 1–5). Overall, the effect of PF-3845 at 3 mg/kg and 10 mg/kg on tissue FAA appeared to be similar with the potential exception of effects at the epicenter.

Table 3 summaries CNS FAA content, as fold-change over vehicle treatment, following PF-3845 treatment in SCI and uninjured rats.

**Table 3:** Fold-change of tissue fatty acid amide levels following a single oral treatment with PF-3845 compared to oral vehicle treatment.

		AEA					OEA					PEA				
Rats	Treatment	Brain	Rostral	Epicenter	Caudal	Lumbar	Brain	Rostral	Epicenter	Caudal	Lumbar	Brain	Rostral	Epicenter	Caudal	Lumbar
SCI	Vehicle	1	1	1	1	1	1	1	1	1	1	1	1	1	1	1
SCI	PF, 3	2.9 (**)	9.8 (***)	4.3 (***)	5.7 (***)	4.6 (***)	5.2 (***)	4.1 (***)	2.7 (**)	3.4 (***)	3.3 (***)	4.5 (***)	3.3 (***)	2.0 (**)	2.6 (***)	2.8 (***)
SCI	PF, 10	3.2 (**)	7.7 (***)	5.9 (***)	6.2 (***)	4.7 (***)	4.9 (***)	3.3 (***)	3.9 (***)	3.8 (***)	3.8 (***)	4.2 (***)	2.6 (***)	2.8 (***)	2.8 (***)	3.1 (***)
N.I.	Vehicle	1	—	1	—	1	1	—	1	—	1	1	—	1	—	1
N.I.	PF, 3	6.4 (***)	—	5.6 (***)	—	5.5 (***)	10.2 (***)	—	3.6 (***)	—	3.7 (***)	10.4 (***)	—	2.6 (***)	—	3.1 (***)
N.I.	PF, 10	5.8 (***)	—	6.0 (***)	—	5.8 (***)	8.9 (***)	—	4.9 (***)	—	3.8 (***)	10.1 (***)	—	3.4 (***)	—	3.5 (***)

Values are fold-changes compared to vehicle treatment levels in spinal cord injured (SCI) and non-injured (N.I) rats. Tissues were collected 4 hours post-oral administration of either PF-3845 (3, 10 mg/kg) or vehicle.

“Epicenter” refers to thoracic spinal cord tissue either from the site of injury from SCI rats or comparable spinal cord tissue from non-injured rats.

*p<0.05, **p<0.01, ***p<0.001 vs. vehicle-treated (Student's t-test).

#### WIN 55,212-2

Interestingly, there were trends towards changes in FAA concentrations following treatment with the nonselective CB receptor agonist WIN 55,212-2 in both uninjured (single treatment) and SCI rats (seven-day treatment). In brain and thoracic spinal cord from uninjured rats, there were tendencies for reduced FAA (Fig. 1–5; Table 2). However, a trend towards an increase in AEA in lumbar spinal cord in uninjured rats was observed following a single treatment of WIN 55,212-2 (*p*>0.05 vs. vehicle treatment).

In SCI rats, there were trends towards both decreased and increased tissue FAA content following WIN 55,212-2 treatment. A trend towards decreased FAA was observed in brain from SCI rats following seven days WIN 55,212-2 treatment (*p*>0.05 vs. vehicle treatment; Table 2). At the same time, spinal cord rostral and caudal to the epicenter showed increased levels of FAA (*p*>0.05 vs. vehicle treatment; Table 2).

### Antinociceptive effect of FAAH inhibitors and WIN 55,212-2 in SCI rats

**Figure 6:**
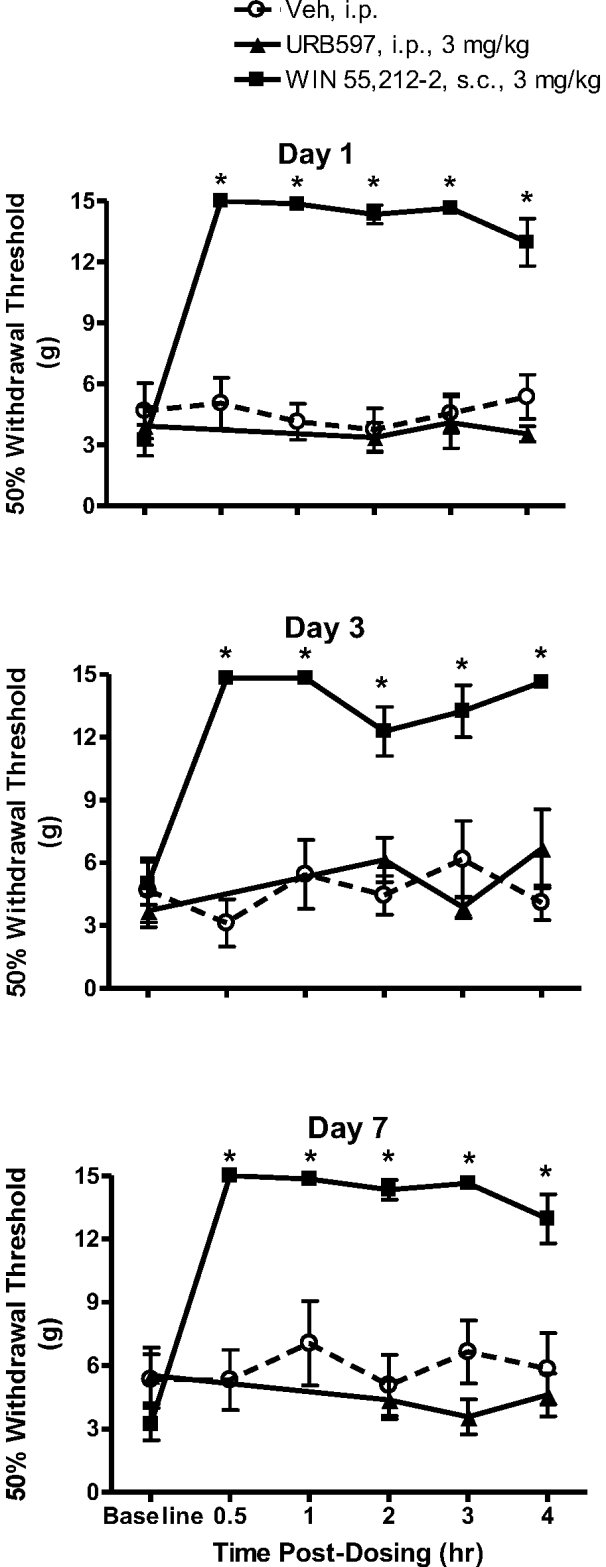
Effects of URB597 and WIN 55,212-2 treatment over seven days on below-level cutaneous hypersensitivity in rats with neuropathic SCI pain. Baseline hind paw withdrawal thresholds were measured prior to treatment with either URB597 (3 mg/kg, i.p.), WIN 55,212-2 (3 mg/kg, s.c.) or vehicle (Veh, 1.5 ml/kg, i.p.). Rats were treated twice daily and tested following the first daily injection. On the first day of testing, a robust antinociception was observed beginning 30 min post-injection of WIN 55,212-2, which was observed also observed on days 3 and 7. By contrast, no antinociceptive effects were observed following treatment with either URB597 or vehicle. Data presented as mean ± S.E.M. n = 8–10/group. * *p*<0.05 vs. vehicle (Two-way repeated measures ANOVA, Student-Newman-Keuls test).

Prior to seven day dosing, the mean hind paw withdrawal threshold of all SCI rats was 3.9±0.6 g (Fig. 6). The mean baseline withdrawal thresholds, within each treatment group, prior to the morning treatment, did not significantly change over the seven day treatment period (*p*>0.05). Significant increases in withdrawal thresholds were observed 30 min following treatment with WIN 55,212-2 on each day of behavioral testing. In fact, on each day of testing, WIN 55,212-2 fully ameliorated hind paw cutaneous hypersensitivity (*p*<0.05 vs. vehicle treatment and vs. baseline; two-way repeated measures-ANOVA). Furthermore, on each of the testing days the effect persisted for the duration of the four hour testing period. By contrast, URB597 did not significantly increase withdrawal thresholds at any time post-treatment (*p*>0.05 vs. vehicle treatment). Likewise, vehicle treatment did not significantly alter withdrawal thresholds.

**Figure 7:**
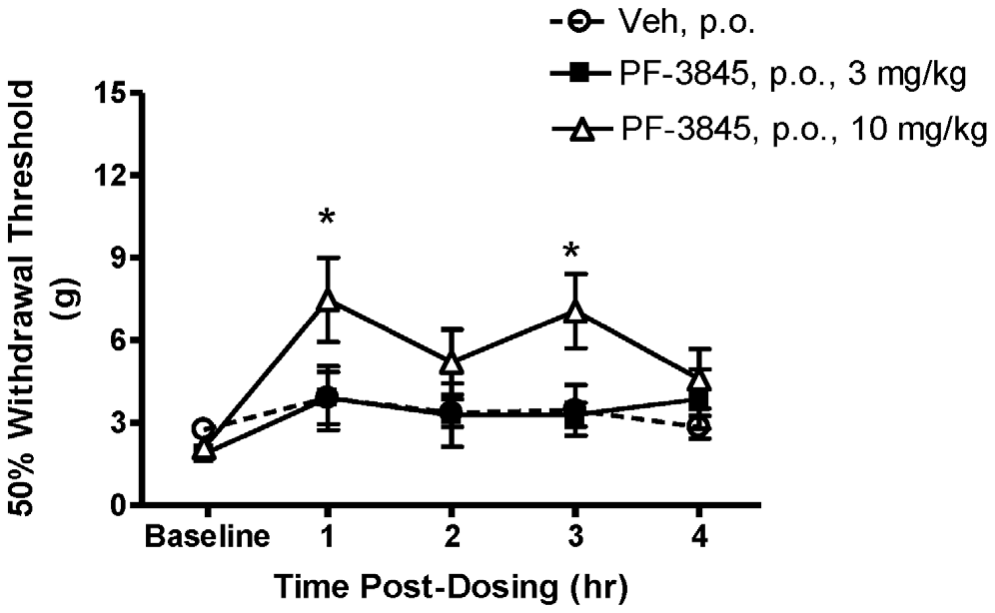
Effect of PF-3845 treatment on below-level cutaneous hypersensitivity in rats with neuropathic SCI pain. Following baseline hind paw withdrawal threshold measurement, SCI rats were treated with either PF-3845 (3, 10 mg/kg, p.o.) or vehicle (Veh; 5 ml/kg, p.o.). An antinociceptive effect was observed 1 and 3 hours following administration of 10 mg/kg, but not 3 mg/kg. Data presented as mean ± S.E.M. n = 8–9/group. * *p*<0.05 vs. vehicle (Two-way repeated measures ANOVA, Student-Newman-Keuls test).

A separate group of SCI rats was dosed once with PF-3845 (Fig. 7). Four weeks following SCI, the mean withdrawal threshold of all rats was 2.3±0.2 g. Increases in withdrawal thresholds were observed with PF-3845 (10 mg/kg) at one and three hours post-treatment (*p*<0.05 vs. vehicle treatment). The increase in withdrawal threshold at one and three hours post-treatment is about a 40% maximum possible effect (100% effect  = 15 g). No significant effects on withdrawal threshold were observed with either 3 mg/kg or vehicle (*p*>0.05).

### Effect of URB597 and WIN 55,212-2 on below-level motor function in SCI rats

**Figure 8:**
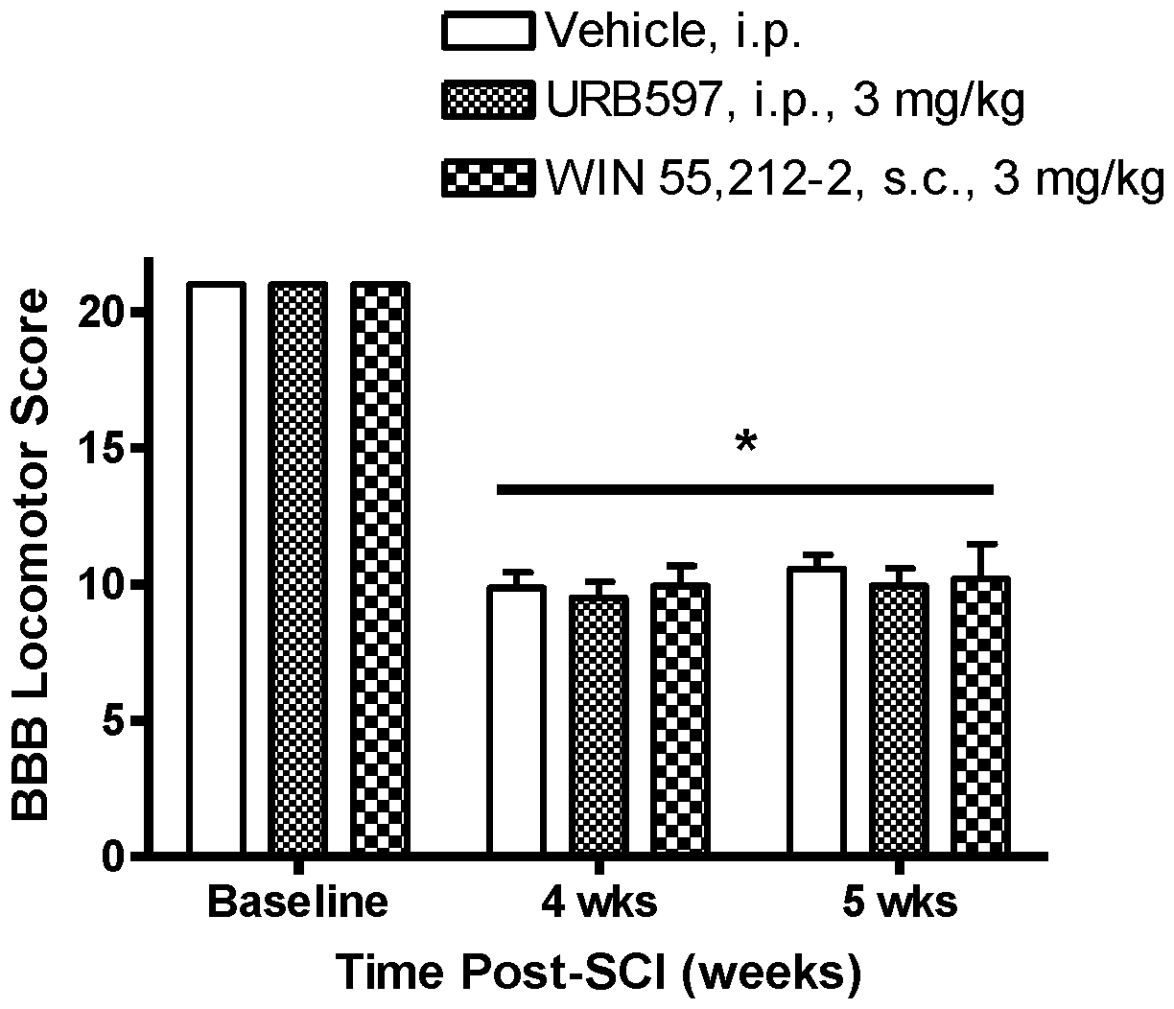
Effects of URB597 and WIN 55,212-2 on BBB Locomotor Scores in SCI rats. Prior to spinal compression surgery (“Baseline”), BBB Locomotor Scores were 21, indicating normal hind limb function. (A Score of “0” indicates no hind limb function.) The mean BBB Locomotor Score obtained the day before initiation of the seven-day treatment procedure (“4 wks” post-SCI) was 9.8±0.4. Spinal cord-injured rats were evaluated again on the sixth day of treatment (“5 wks” post-SCI) and there were no significant changes in BBB Scores. Data presented as mean ± S.E.M. n = 8–10/group. *p<0.05 vs. pre-SCI (Two-way repeated measures ANOVA, Student-Newman-Keuls test).

Prior to treatment, the mean BBB Locomotor Scores of all SCI rats was significantly decreased from 21 to 9.8±0.4 (*p*<0.05; Fig. 8). This score means frequent stepping with the dorsal rather than the plantar hind paw and no fore limb-hind limb coordination. After six days of treatment with either URB597, WIN 55,212-2 or vehicle, BBB Locomotor Scores were 9.9±0.6, 10.2±1.3, 10.6±0.5, respectively (*p*>0.05 vs. vehicle treatment). Compared with BBB Scores before treatment, BBB Scores were not significantly changed after six days of treatment in SCI rats (*p*>0.05; Fig. 8). The effect of a single dose of PF-3845 on BBB Locomotor Scores was not assessed.

## Discussion

Despite significantly increasing brain and spinal cord tissue levels of FAA in SCI rats, seven day systemic administration of the FAAH inhibitor URB597 did not demonstrate antinociception. Acute treatment with the FAAH inhibitor PF-3845, which also significantly increased CNS levels of FAA, led to a moderate, non-dose dependent antinociception in SCI rats. By contrast, WIN 55,212-2 robustly ameliorated below-level cutaneous hypersensitivity in SCI rats for the duration of the seven-day treatment period. Markedly decreased tissue levels of OEA and PEA were observed at the epicenter and tissue caudal to the epicenter in SCI rats. While both FAAH inhibitors increased tissue FAA in SCI rats, only PF-3845 increased AEA, OEA and PEA at the epicenter, which in part could underlie PF-3845's modest antinociceptive effect. Neither FAAH inhibition nor CB receptor activation over seven days improved hind limb motor function. While direct activation of CB receptors leads to a significant and sustained antinociception, the current data indicate that increasing CNS FAA levels alone may not be sufficient for ameliorating below-level neuropathic SCI pain.

Changes in FAA levels in spinal tissue segments that may be crucial in the initiation and maintenance of neuropathic pain following a SCI were reported in the current study. The current study confirmed a previous finding of no change in levels of AEA and PEA in spinal tissue rostral to the epicenter and at the epicenter four weeks after a SCI [26]. However, the current study observed that PEA was decreased caudal to the epicenter and that OEA was decreased both rostrally and caudally to the epicenter as well as within the epicenter. It is speculated that the loss of tonic FAA-meditated inhibition following SCI could contribute to enhanced excitability of spinal dorsal horn neurons either rostral or caudal to the epicenter [2], [27]. In the current study, slight decreases (*p*>0.05; Table 1) in FAA were observed in the lumbar spinal cord, which receives primary afferents that innervate the hind paw. The mechanism that leads to an apparent decrease of FAA in lumbar segment, several segments from the epicenter, has yet to be elucidated, but a consequence could be dorsal horn neuron hyperexcitability. However, lumbar spinal cord FAA were significantly increased following URB597 treatment without being accompanied by significant antinociception. Likewise, FAA levels in lumbar spinal cord were increased following PF-3845, but only a slight antinociceptive effect was obtained. Convincing evidence that the loss of CB_1_ receptor-mediated tonic inhibition alone can lead to hyperexcitable lumbar dorsal horn neurons is lacking, but perhaps it is a fusion of diminished functioning of several neurotransmitter systems (e.g. GABA) that leads to the neuropathic pain state following SCI [28], [29].

Changes in supraspinal function could also be crucial in the maintenance of neuropathic SCI pain [16], [19]. The current study found no significant change in whole brain FAA levels following SCI, but perhaps there are changes in specific pain-related brain nuclei following SCI. It is further speculated that processes involved in cellular function and intercellular signaling could be altered in the brain as well as in the spinal cord following injury [30], [31]. Changes in the brain opioid system have been suggested to underlie reduced efficacy of supraspinal injection of morphine in SCI rats [32], [33]. By contrast, supraspinal injection of WIN 55,212-2 is fully efficacious in SCI rats [19]. Changes in either brain CB receptors or FAA level have yet to be identified following SCI. Nonetheless, despite a robust increase in whole brain FAA following FAAH inhibition, there was a lack of robust antinociception; a role for brain FAA in SCI pain will need further elucidation.

A number of preclinical neuropathic pain studies have demonstrated marked antinociception following both acute and repeated activation of CB receptors, which appears to be in part mediated through the CB_1_ receptor [3], [20], [34]–[38]. It is possible that attenuation of specific symptoms, such as hypersensitivity to noxious stimulation (hyperalgesia), could be ameliorated through other CB receptors or non-CB receptors [11], [39].

The current study confirms previous findings in neuropathic pain models of a lack tolerance to the antinociceptive effect following repeated treatment with a CB receptor agonist [20], [35], [36]. By contrast, a loss of efficacy following repeated treatment with morphine in SCI rats has been observed [20]. Thus, a potential advantage of CB1 receptor agonists over, for example, opioid receptor agonists is sustained efficacy over the course of the treatment period. Tolerance to CB receptor agonists does appear however, under specific circumstances. In the case of an acute pain state, as modeled by the hot plate test in uninjured animals, tolerance to the antinociceptive effect appears within five days of daily treatment [20], [40]. The mechanism by which CB receptor agonists retain efficacy in some pain states and not in others is currently unknown. While it is possible that the injury state could underlie the development of antinociceptive tolerance, the test stimulus could also influence whether or not tolerance is obtained.

One issue raised by CB receptor agonists is the presence of motoric effects at or near antinociceptive doses. In the case of WIN 55,212-2, the 50% antinociceptive dose is 0.9 mg/kg (s.c., 30 min post-dosing) whereas the dose needed to produce 50% disruption on the rotarod test (s.c., 3 hrs. post-dosing) is about the same [14], [20]. In a previous study, a sedating dose of the anxiolytic drug diazepam, did not increase hind paw withdrawal thresholds in SCI rats, indicating that the behavioral endpoint is sensitive to antinociceptive treatment rather than to a treatment that merely disrupt motor function [18]. Thus, the increase in withdrawal thresholds observed with WIN 55,212-2 in the current study cannot be entirely attributed to catalepsy or motor dysfunction.

While targeting CB_1_ receptors is a promising therapeutic strategy for ameliorating neuropathic SCI pain, prominent psychomotor side effects are associated with CB_1_ receptor activation [14], [20]. Alternatively, elevating synaptic levels of FAA is antinociceptive and does not appear to induce adverse side effects as the antinociception appears to be mediated through the CB_2_ receptor, and possibly through other non-CB mediated mechanisms, such as through activation of the TRPV1 channel, as well as through the CB_1_ receptor [9]–[11], [41]. URB597 is a potent FAAH inhibitor—50% of brain FAAH activity is inhibited at a dose of 0.15 mg/kg (i.p.) [10]. At doses higher than 0.3 mg/kg, 1 and 3 mg/kg (i.p.), levels of FAA do not appear to be greatly increased, thus a FAAH inhibition plateau is reached at about 1 mg/kg [42]. In vivo, URB597 inhibits the degradation of AEA within 15 min of injection and significant inhibition of FAAH activity is observed up to 12 hours post-injection [10]. Importantly, no deleterious effect on motor function, as assessed in the rotarod test, was observed at doses of up to 5 mg/kg of URB597 and robust behavioral effects (e.g. antinociception, anxiolytic-like effects) were observed at very low doses (0.1–0.5 mg/kg, i.p.) [13], [43]. In the current study, significant spinal tissue levels of FAA were obtained with 3 mg/kg URB597 in both SCI and uninjured rats. Similarly, both doses of PF-3845 significantly raised FAA levels in spinal tissues, including the epicenter of spinal cord from SCI rats as well as spinal tissue from uninjured rats. Thus, the current study demonstrates that it is possible to increase FAA in injured spinal tissue.

Despite significantly increasing CNS FAA levels, no antinociceptive effect was observed with URB597 in SCI rats, following either a single dose (day 1) or multiple doses (at either day 3 or day 7). A single dose of URB597 in peripheral nerve-injured mice resulted in a small antinociceptive effect, but four days of treatment, however, led to an even greater antinociceptive effect [22]. One significant difference between the previous studies showing efficacy with URB597 and the current study is that previous studies utilized models of peripheral neuropathy whereas the current study utilized a model of central pain following a SCI. There are a number of drugs that are efficacious in peripheral neuropathic pain models but are not efficacious in neuropathic SCI models [17], [18]. Why this is so is not entirely clear, but the differential pharmacology between peripheral and central neuropathic pain states highly suggests that there are meaningful differences in the substrates that mediate the symptoms presented by these pain states. The differential substrates hypothesized to exist in preclinical models is indirectly supported by clinical findings, as a similar pharmacological divergence also exists between clinical neuropathic SCI and peripheral neuropathic pains [2].

Whereas URB597 lacked efficacy, PF-3845 led to a partial, non-dose dependent amelioration of neuropathic SCI pain. In addition to inhibitory effects on neurons, FAA concurrently suppress the release of pro-inflammatory mediators from immune cells and facilitate the release of anti-inflammatory substances [26]. It is possible that PF-3845 is more potent than URB597 on FAAH expressed in microglia and macrophage or that PF-3845 better penetrates damaged tissue as found at the epicenter to reach these cell types. In fact, 3 mg/kg (p.o.) PF-3845 led to a greater increase of AEA in CNS tissues, including the epicenter, compared to 3 mg/kg (i.p.) URB597. It has been reported elsewhere that FAA levels in mice treated with PF-3845 were higher compared to mice treated with URB597, despite the same dose and route [12]. Following PF-3845 treatment, FAA and anti-inflammatory substances from the epicenter could diffuse to nearby spinal dorsal horn neurons that are hyperexcited following SCI [44]. Perhaps it is the increase in anti-inflammatory substances within and adjacent to the epicenter that is crucial in suppressing hyperexcited central neurons. The antinociceptive effect observed with PF-3845 in neuropathic SCI rats could be partially mediated through a combination of CB receptor-dependent and CB receptor-independent mechanisms [45], [46].

While no robust effect was observed on below-level pain in the current study, it is possible that above-level and at-level SCI pain could be attenuated by FAAH inhibition. In addition to significant hyperexcitation of central neurons, there appears to be significant primary afferent hyperexcitation following SCI, which could underlie cutaneous hypersensitivity and spontaneous pain at and above the SCI [44], [47], [48]. These afferents could be sensitive to FAA through CB receptor or non-CB receptor mediated mechanisms or both. Increased sodium channel expression has been observed in injured peripheral nerves following injury—perhaps a similar increase in sodium channel expression in peripheral nerves following a SCI, as reported in the CNS, underlies at-level pain [49], [50]. Indeed, SCI patients have reported relief from at-level pain following application of either capsaicin, a TRPV1 agonist, or lidocaine, a sodium channel blocking local anesthetic [51], [52]. Perhaps the slight increase in FAA obtained with PF-3845 would have been sufficient to reduce at-level neuropathic pain. Further studies, however, are needed to characterize the extent of the involvement of primary afferent neurons in SCI pain.

A closer examination of studies that tested FAAH inhibitors on peripheral neuropathic cutaneous hypersensitivity indicates weak to no efficacy. As mentioned earlier, a single treatment of URB597 (10 mg/kg, p.o.) in mice with a chronic constriction injury of the sciatic nerve led to a small antinociceptive effect [22]. In rats with a spinal nerve ligation, URB597 (0.3 mg/kg, i.p.) treatment led to a 42% reversal of hind paw tactile hypersensitivity [42]. It is possible that higher doses of URB597 could yield greater antinociception, even though Karbarz et al. (2009) reported no further increases in FAA with higher doses. In rats with a partial sciatic nerve ligation, no effect was obtained with URB597 (0.3 mg/kg, i.p.), but the same dose was significantly efficacious in rats with a peripheral inflammation [13]. Likewise, significant antinociception and brain FAA levels were obtained in rats with a hind paw inflammation at 3 mg/kg PF-3845 (p.o.) [12]. In FAAH deficient mice, brain AEA levels were about 10-fold higher than that of wild-type mice [53]. Following a sciatic nerve injury, FAAH knockout mice demonstrated marked ipsilateral hind paw hypersensitivity, indicating that increased CNS AEA did not prevent the onset of nerve injury-induced nociception. However, the degree of hind paw carrageenan-induced hypersensitivity in FAAH deficient mice was not as severe as that seen in wild-type mice. While the findings indicate some antinociception following increased CNS FAA in the neuropathic state, the antinociception is not as robust as compared to that obtained in the painful inflammatory state. It is speculated that a SCI that is accompanied by significant inflammation could be sensitive to the effect of FAAH inhibition. Perhaps early treatment, around the time of injury or soon thereafter, will be efficacious.

A potential limitation of the current study is the dose of URB597 used. Perhaps a much greater dose of URB597 would have shown efficacy, even though it appears that there is no further gain in FAA levels in the CNS [42]. In mice with a peripheral nerve injury antinociceptive effects of URB597 were observed at 10 mg/kg (i.p.) and at 50 mg/kg (p.o.) [22], [54]. The antinociceptive effects were attenuated with CB_1_ receptor antagonist, indicating mediation through CB_1_ receptors. Interestingly, Russo et al. (2007) saw only partial attenuation of the effect of URB597 with CB_2_ receptor antagonist treatment whereas Kinsey et al. (2009) observed full attenuation. The different behavioral endpoints and potential off-target activity of a high dose could in part explain the differences between the two findings. Nonetheless, perhaps much higher doses of URB597 in the SCI rats could lead to robust antinociception.

A recent clinical study of the FAAH inhibitor PF-04457845 in osteoarthritis pain [55] showed a lack of analgesic efficacy despite demonstration of significant elevation of FAA in patients' plasma and significant efficacy in preclinical studies in rodent models of non-neuropathic pain [56]. The disconnection between the preclinical findings and clinical outcomes raises issues of translatability, of whether clinical experimental methods in fact parallel preclinical experimental methods and whether preclinical models accurately model the clinical state. On one hand, it is not entirely clear if peripherally measured levels of the biomarker of interest are adequate in order to make predictions on behaviors that are CNS mediated. On the other hand, there may be a tenuous relationship of some preclinical animal models (e.g. the rat hind paw formalin test) to long-standing pathologies such as osteoarthritis. The current study utilized a rat model of neuropathic SCI pain, in which analgesics used for clinical neuropathic SCI pain appear antinociceptive [18]. The current findings suggest that increasing FAA alone may not be an efficacious treatment specifically for below-level SCI neuropathic pain.

While the current study suggests at most a weak antinociceptive effect of FAAH inhibition alone, greater antinociceptive effects could be obtained by combining an FAAH inhibitor with a known analgesic drug, thereby enhancing the efficacy of the analgesic drug [57]. The combination of opioids with AEA leads to a synergistic antinociception [58], [59]. A metabolite of acetaminophen has been shown to enhance synaptic AEA by blocking its transporter [60]. Although acetaminophen alone is not antinociceptive in SCI rats, adding it to analgesic drugs led to synergistic antinociception which was CB_1_ receptor-mediated [61]. Combination drug therapy, with FAAH inhibitors at the core, could be of great value for neuropathic SCI pain, for which few effective analgesic treatments exist [9], [62].
